# Novel Type of *Streptococcus pneumoniae* Causing Multidrug-Resistant Acute Otitis Media in Children

**DOI:** 10.3201/eid1504.071704

**Published:** 2009-04

**Authors:** Qingfu Xu, Michael E. Pichichero, Janet R. Casey, Mingtao Zeng

**Affiliations:** University of Rochester School of Medicine and Dentistry, Rochester, New York, USA (Q. Xu, M.E. Pichichero, M. Zeng); Legacy Pediatrics, Rochester (M.E. Pichichero, J.R. Casey)

**Keywords:** Antimicrobial resistance, streptococcal diseases, acute otitis media, multilocus sequence typing, Streptococcus pneumoniae, serotypes, research

## Abstract

After our recent discovery of a *Streptococcus pneumoniae* 19A “superbug” (Legacy strain) that is resistant to all Food and Drug Administration–approved antimicrobial drugs for treatment of acute otitis media (AOM) in children, other *S. pneumoniae* isolates from children with AOM were characterized by multilocus sequence typing (MLST). Among 40 isolates studied, 16 (40%) were serotype 19A, and 9 (23%) were resistant to multiple antimicrobial drugs. Two others had unreported sequence types (STs) that expressed the 19A capsule, and 8 (88%) of the 9 multidrug-resistant strains were serotype 19A, including the Legacy strain with the new ST-2722. In genetic relatedness, ST-2722 belonged to a cluster of reported strains of *S. pneumoniae* in which all strains had 6 of the same alleles as ST-156. The multidrug-resistant strains related to ST-156 expressed different capsular serotypes: 9V, 14, 11A, 15C, and 19F.

## Abstract 2

A new multidrug-resistant strain of serotype 19A has been characterized in upstate New York.

The increasing global emergence and rapid spread of multidrug-resistant *Streptococcus pneumoniae* is a serious concern (*1**,**2*). Clonal dissemination of problematic pneumococcal strains have created clinically important treatment problems (*3**,**4*). Pneumococcal resistance to antimicrobial drugs was first reported in the mid-1960s (*5**,**6*). Since 1990, drug-resistant isolates of *S. pneumoniae* have spread rapidly throughout the United States (*7*). In the early 1990s, high-level resistance to penicillin and other antimicrobial drugs appeared in the United States with a low prevalence (*8*). Over the past decade, multidrug-resistant clones of *S. pneumoniae* have rapidly emerged (*8**–**11*). Of 90 serotypes, 19A is one of the most common types found in children. It can cause severe disease and easily develop antimicrobial drug resistance (*9*). The first 19A strain of *S. pneumoniae* with penicillin resistance was reported in the United States in 1986 (*12*). Introduction in 2000 of a pneumococcal 7-valent pneumococcal conjugate vaccine (PCV7) in the United States has substantially curtailed pneumococcal infections caused by 7 vaccine strains in children (*13**–**15*). However, nonvaccine strains, including drug-resistant strains, are increasingly being identified in patients. Among multidrug-resistant strains, a high proportion is serotype 19A, a strain not included in the vaccine; however, the proportion of invasive pneumococcal diseases caused by serotype 19A has substantially increased (*8**–**11*).

We recently discovered and reported a “superbug” strain of *S. pneumoniae* (Legacy strain) that is resistant to all Food and Drug Administration (FDA)–approved antimicrobial drugs and to 8 non-FDA–approved antimicrobial drugs used to treat acute otitis media (AOM) in children (*11*). Using molecular epidemiologic methods, particularly multilocus sequence typing (MLST), we characterized the molecular type of multidrug-resistant strains of *S. pneumoniae* recently isolated from children with AOM, compared the Legacy strain sequence type (ST) 2722 with 67 strains with the closest types in the MLST database, and reported the likely evolution and spread of the ancestor strains of the Legacy strain.

## Methods

### Pneumococcal Isolates, Serotype Analysis, and Antimicrobial Drug–Susceptibility Testing

All pneumococcal bacteria were isolated from children seen at a private pediatric group in suburban Rochester, New York (Legacy Pediatrics). Isolates were obtained from middle ear fluid of children with AOM at 6–36 months of age during 2004–2006. The children received age-appropriate inoculations with PCV7 at 2, 4, 6, and 12–15 months of age. The University of Rochester Institutional Review Board approved the study, and written informed consent was obtained from parents or guardians for the study and for all tympanocentesis procedures. Serotypes of pneumococci were determined by latex agglutination (Pneumotest-Latex; Statens Serum Institute, Copenhagen, Denmark) according to the manufacturer's instructions and confirmed by Quelling reaction. The antimicrobial drug susceptibility of pneumococci was determined as described previously by E-test or microbroth dilution (*11*).

### Multilocus Sequence Typing

The internal fragments of 7 housekeeping genes (*aroE* [shikimate dehydrogenase], *gdh* [glucose-6-phosphate dehydrogenase], *gki* [glucose kinase], *recP* [transketolase], *spi* [signal peptidase I], *xpt* [xanthine phosphoribosyltransferase], and *ddl* [D-alanine-D-alanine ligase]) were amplified from chromosomal DNA by PCR. Chromosomal DNA was extracted from subculture of *S. pneumoniae* isolations recovered from middle ear fluid, nasal wash, or nasal swabs. PCR amplification was performed using primer pairs *aroE*-up, 5′-GCCTTTGAGGCGACAGC-3′ and *aroE*-dn, 5′-TGCAGTTCA(G/A)AAACAT(A/T)TTCTAA-3′; *gdh*-up, 5′-ATGGACAAACCAGC (G/A/T/C)AG(C/T)TT-3′ and *gdh*-dn, 5′-GCTTGAGGTCCCAT(G/A)CT(G/A/T/C) CC-3′; *gki*-up, 5′-GGCATTGGAATGGGATCACC-3′ and *gki*-dn, 5′-TCTCCCGCAGCTGACAC-3′; *recP*-up, 5′-GCCAACTCAGGTCATCCAGG-3′ and *rec*P-dn, 5′-TGCAACCGTAGCATTGTAAC-3′; and *spi*-up, 5′-TTATTCCTCCTGATTCTGTC-3′ and *spi*-dn, 5′-GTGATTGGCCAGAAGCGGAA-3′. PCR conditions were as follows: initial denaturation at 95°C for 5 min, followed by 30 cycles of 95°C for 30 s; annealing at 50°C–55°C for 30 s; and extension at 72°C for 30 s. The amplified DNA fragments were purified by using QIAquick PCR Purification Kit (QIAGEN, Valencia, CA, USA) and were sequenced in each direction by using the same primers used for amplification and by using the BigDye Terminator v3.1 Cycle Sequencing Kit on an Applied Biosystems Prism 377 automated sequencer (Applied Biosystems, Foster City, CA, USA).

The sequences at each of the 7 loci were then compared with the sequences of all of the known alleles at those loci in the database at the pneumococcal MLST website (http://spneumoniae.mlst.net). The sequences identical to a known sequence were assigned the same allele number, and nonidentities to any known allele sequences were assigned new allele numbers. The allele at each of the 7 loci defines the allelic profile of each strain, as well as its ST. New allelic number or new ST number was assigned by a curator of the pneumococcal MLST database. The relatedness of isolates and known similar strains in the database was determined by constructing a neighbor-joining tree using a program online, Draw Tree Using Own MLST Data, found at the pneumococcal MLST website.

## Results

Among 40 *S. pneumoniae* isolates recovered from middle ear fluid of children with AOM, 16 (40%) were serotype 19A. Serotype 19A was the most common serotype isolated in the study during 2004–2006.

Antimicrobial drug susceptibility testing was performed on the serotype 19A isolates. Eight (50%) of the 16 isolates of 19A were highly penicillin resistant (MIC >2.0 µg/mL), and all 8 were also multidrug resistant.

**Figure 1:**
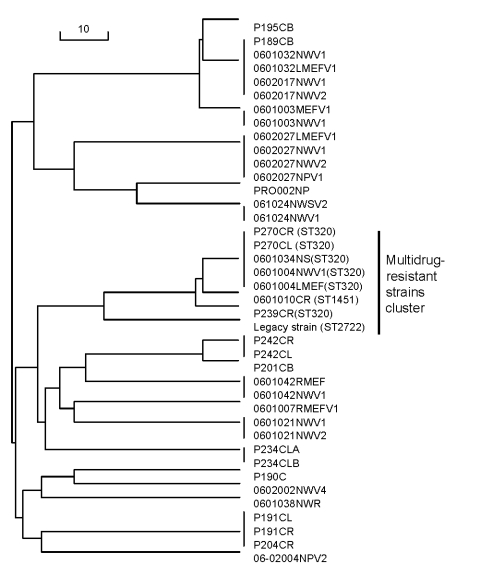
Neighbor-joining tree of genetic relatedness among 40 *Streptococcus pneumoniae* isolates from a private pediatric practice, Rochester, New York, USA, 2004–2006. Scale bar indicates genetic linkage distance.

MLST of these16 *S. pneumoniae* isolates with serotype 19A showed that 6 (38%) were ST-320; 3 (19%) were ST-199; 2 (13%) were unreported STs now assigned ST- 2722 and ST-2704; 2 (13%) were ST-1673; and 1 (6%) each of the remaining 3 was ST-1451, ST-2265, and ST-63. Among the 8 multidrug-resistant isolates, 5 (63%) were ST-320, 1 (13%) was ST-1451, and 1 (13%) was the newly assigned ST-2722 (Table 1, Figure 1). The genetic distance of the Legacy strain is closest to 7 other multidrug-resistant strains with ST-320 and ST-1451 in our group of 40 recent isolates.

**Table 1:** Genotypic characteristics of *Streptococcus pneumoniae* serotype 19A strains with multiple antimicrobial drug resistance*

Strain	Sequence type	MIC, µg/mL					Allele no. of housekeeping genes†						
		Pen	Cef	Ery	S.Tri		*aroE*	*gdh*	*gki*	*recP*	*spi*	*xpt*	*ddl*
P270CR	320	2.0	1.0	>256	>32		4	16	19	15	6	20	1
060110	1451	4.0	1.0	>256	4.0		10	16	19	15	6	20	1
P270CL	320	2.0	1.0	>256	>32		4	16	19	15	6	20	1
Legacy strain	2722‡	8.0	6.0	8.0	>32		7	11	10	15	6	8	1
P239CR	320	2.0	2.0	>256	8.0		4	16	19	5	6	20	1
0601034	320	2.0	1.0	>256	16.0		4	16	19	15	6	20	1
0601004V1NW	320	2.0	1.5	>256	6.0		4	16	19	15	6	20	1
0601004V1LMEF	320	3.0	1.5	>256	4.0		4	16	19	15	6	20	1

*Pen, benzylpenicillin; Cef, ceftriaxone; Ery, erythromycin; S.Tri, sulfamethoxazole-trimethoprim. †*aroE* (shikimate dehydrogenase), *gdh* (glucose-6-phosphate dehydrogenase), *gki* (glucose kinase), *recP* (transketolase), *spi* (signal peptidase I), *xpt* (xanthine phosphoribosyltransferase), and *ddl* (D-alanine-D-alanine ligase). ‡New assigned sequence type.

**Figure 2:**
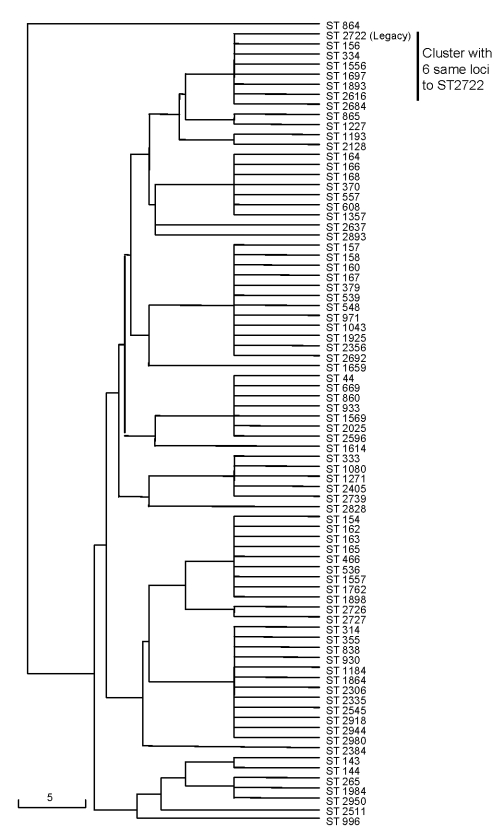
Genetic relatedness of *Streptococcus pneumoniae* ST-2722 (Legacy strain) to reported types that have 5 or 6 of the same loci as the Legacy strain. List of types available at the multilocus sequence typing database (http://spneumoniae.mlst.net). Scale bar indicates genetic linkage distance.

**Figure 3:**
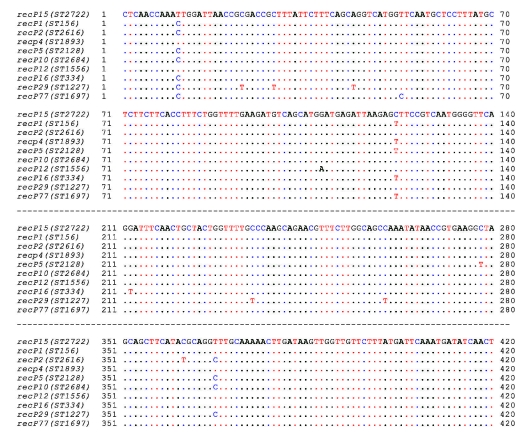
Variable sites (colors) in *recP* DNA sequences (allele) of ST-2722 (Legacy strain) and those in other *Streptococcus pneumoniae* that have the same 6 loci. List of types available at the multilocus sequence typing database (http://spneumoniae.mlst.net).

To determine the evolutionary relationship of the Legacy strain (ST-2722) to other strains, we constructed a neighbor-joining tree. ST-2722 is assumed to have genetic relatedness to another ST in the database when an ST has >5 of the same loci as ST-2722 (Figure 2). ST-2722 belonged to a cluster in which all strains have 6 of 7 same loci. This cluster consisted of 59 (88%) strains with molecular type ST-156 and 1 strain each with ST-2722, ST-2128, ST-1227, ST-2616, ST-1893, ST-334, ST-1556, ST-2684, and ST-1697. The major dissimilarity of strains in this cluster was on loci *recP* (Table 2, Figure 3). The *recP* gene had 3 major variable sites at the 10th, 121st, and 368th bp among strains. For instance, the DNA at the 10th, 121st, and 368th bp are T, C, T, respectively, in ST-2722; C, T, T in ST-156; and C, T, C in ST-2128 (Figure 3).

**Table 2:** Comparison of *Streptococcus pneumoniae* ST-2722 (Legacy strain) with the closest types that have 6 of the same alleles as ST-2722*

ST	Serotypes	Allele profile of housekeeping genes†							No. resistant/ total strains	Countries of isolation	Sources
		*aroE*	*gdh*	*gki*	*recP*	*spi*	*xpt*	*ddl*			
156	9V/14/11A/ 15C/19F	7	11	10	1	6	8	1	38/59	BR, CA, CZ, DK, FR, DE, HU, IL, IT, LB, NL, PL, SE, PT, QA, ES, UK, UY	Nasopharynx, cerebrospinal fluid, blood, pleural, sputum, ear
1556	14	7	11	10	12	6	8	1	1/1	DE	Blood
1697	9V	7	11	10	77	6	8	1	1	UK	NA
2684	11A	7	11	10	10	6	8	1	1/1	FR	Nasopharynx
334	9	7	11	10	16	6	8	1	1/1	NO	Blood
1893	14	7	11	10	4	6	8	1	1	NO	NA
2616	14	7	11	10	2	6	8	1	1	ES	Nasopharynx
1227	14	7	11	10	29	6	8	1	1/1	SE	Nasopharynx
2128	9	7	11	10	5	6	8	1	1/1	UK	Blood
2722	19A	7	11	10	15	6	8	1	1	US	Ear

*Types reported in the multilocus sequence typing database (http://spneumoniae.mlst.net). ST, sequence type; BR, Brazil; CA, Canada; CZ, Czech Republic; DE, Germany; DK, Denmark; FR, France; HU, Hungary; IL, Israel; IT, Italy; LB, Lebanon; NL, Netherlands; NO, Norway; PL, Poland; SE, Sweden; PT, Portugal; QA, Qatar; ES, Spain; UK, United Kingdom; UY, Uruguay; US, United States; NA, not available. †*aroE* (shikimate dehydrogenase), *gdh* (glucose-6-phosphate dehydrogenase), *gki* (glucose kinase), *recP* (transketolase), *spi* (signal peptidase I), *xpt* (xanthine phosphoribosyltransferase), and *ddl* (D-alanine-D-alanine ligase).

Forty-three (64%) of the 67 strains related to ST-2722 were penicillin resistant, and 28 (42%) of these strains were resistant to at least 1 other antimicrobial drug. Thirty-four (51%) of the 67 strains related to ST-2722 serotype 19A strains were serotype 9V; 18 (27%) were serotype 14; and 5 (7%) were serotype 11A. Among the 43 antimicrobial drug–resistant strains in this cluster, 24 (56%) were serotype 9V, and 14 (33%) were serotype 14; others were serotype 11, 15C, and 19F.

## Discussion

We described the molecular and capsular types of pneumococci causing AOM in children tested in Rochester, New York, during 2004–2006. Serotype 19A, a relatively new molecular type, accounted for 40% of the isolates and has emerged as the major serotype causing ear infections in children. Eight different molecular STs expressed the 19A capsule; most of the strains were multidrug resistant. Among the 40 strains studied, 5% were new molecular STs. In addition, we analyzed the genetic relatedness of the strains to other previously described strains of serotype 19A. We traced the genetic origin of ST-2722 to a strain first identified in 1988 in Spain as ST-156, which expressed capsular serotype 9V (*16*). Over nearly 20 years before ST-2722 emerged, variants of this original ST-156 strain were identified in 18 countries, with 8 different STs and 13 different sequence/capsular type combinations (according to records in *S. pneumoniae* database).

ST-2722 (Legacy strain) appears to have resulted from a mutation identified in the *recP* gene that coincided with acquisition of multidrug resistance, including a ceftriaxone MIC of 6.0 µg/mL, and acquisition of capsular DNA associated with the 19A serotype. Because of genetic plasticity, different capsular genes of *S. pneumoniae* may transfer through DNA-mediated genetic recombination (*17**–**19*). The multidrug-resistant epidemic type 23F Spanish/USA clone has expressed capsular types 3, 9N, 14, and 19F. Capsular transformation may equip multidrug-resistant strains with highly virulent blood invasive phenotypes (*18*).

Many studies have documented the impact of the PCV7 on pneumococcal diseases. Introduction of PCV7 containing serotype 4, 6B, 9V, 14, 18C, 19F, and 23F has dramatically decreased the rate of carriage and disease caused by these vaccine serotypes. However, the proportion of invasive pneumococcal diseases caused by nonvaccine serotypes has increased substantially in recent years, and multiresistant strains have rapidly emerged (*16**,**20**–**23*). In addition, PCV7 offers moderate protection against AOM (*24*). The lowest level of protective activity provided by PCV7 was against serotype 19F, and 19F polysaccharide antigen provided less cross-protection for disease caused by related serotypes such as 19A (*24**,**25*). An increase in the rate of middle ear infections with 19A strains and other strains expressing capsular types not contained in PCV7 also has been reported (*11*). These developments encourage further ongoing epidemiologic surveillance. Emergence of new 19A strains may represent a successful vaccine escape mechanism used by PCV7-targeted clones, and antimicrobial drug nonsusceptibility provides an additional survival advantage that could help these organisms spread further.

By MLST analysis and capsular typing, we found that multiple STs expressing capsular type 19A have emerged as the most important otopathogens of children. We also found a new strain of *S. pneumoniae* (ST-2722)*,* expressing a 19A capsule that is resistant to all FDA-approved antimicrobial drugs. ST-2722 has a genetic relatedness close to ST-156 reported to the *S. pneumoniae* MLST database from 18 countries. ST-2722 was multiresistant to antimicrobial drugs but had an MIC to ceftriaxone of 6.0 µg/mL. A study by Pelton et al. showed that multidrug-resistant 19A strains with ST-320 had an MIC to ceftriaxone of 8.0 µg/mL (*10*). The clinical importance of such strains is potentially high because empiric treatment of suspected and even proven pneumococcal infections typically relies on the efficacy of ceftriaxone.
